# Revisiting the radiographic assessment of osteoporosis—Osteopenia in children 0–2 years of age. A systematic review

**DOI:** 10.1371/journal.pone.0241635

**Published:** 2020-11-02

**Authors:** Karen Rosendahl, Anette Lundestad, John Asle Bjørlykke, Regina Küfner Lein, Oskar Angenete, Thomas Angell Augdal, Lil-Sofie Ording Müller, Diego Jaramillo

**Affiliations:** 1 Faculty of Health Sciences, Department of Clinical Medicine, UiT the Arctic University of Norway, Tromsø, Norway; 2 Section of Paediatric Radiology, University Hospital of North Norway, Tromsø, Norway; 3 Department of Paediatrics, St. Olav Hospital, Trondheim, Norway; 4 Section of Paediatric Radiology, Haukeland University Hospital, Bergen, Norway; 5 University Library, Bergen University, Bergen, Norway; 6 Department of Radiology and Nuclear Medicine, St Olav Hospital, Trondheim, Norway; 7 Department of Circulation and Medical imaging, Faculty of Medicine, Norwegian University of Science and Technology, Trondheim, Norway; 8 Section of Paediatric Radiology, Oslo University Hospital, Oslo, Norway; 9 Columbia University Medical Center, New York, New York, United States of America; Medical College of Wisconsin, UNITED STATES

## Abstract

**Background:**

Imaging for osteoporosis has two major aims, first, to identify the presence of low bone mass (osteopenia), and second, to quantify bone mass using semiquantitative (conventional radiography) or quantitative (densitometry) methods. In young children, densitometry is hampered by the lack of reference values, and high-quality radiographs still play a role although the evaluation of osteopenia as a marker for osteoporosis is subjective and based on personal experience. Medical experts questioned in court over child abuse, often refer to the literature and state that 20–40% loss of bone mass is warranted before osteopenia becomes evident on radiographs. In our systematic review, we aimed at identifying evidence underpinning this statement. A secondary outcome was identifying normal references for cortical thickness of the skeleton in infants born term, < 2 years of age.

**Methods:**

We undertook systematic searches in Medline, Embase and Svemed+, covering 1946–2020. Unpublished material was searched in Clinical trials and International Clinical Trials Registry Platform (ICTRP). Both relevant subject headings and free text words were used for the following concepts: osteoporosis or osteopenia, radiography, children up to 6 years.

**Results:**

A total 5592 publications were identified, of which none met the inclusion criteria for the primary outcome; the degree of bone loss warranted before osteopenia becomes visible radiographically. As for the secondary outcome, 21 studies were identified. None of the studies was true population based and none covered the pre-defined age range from 0–2 years. However, four studies of which three having a crossectional and one a longitudinal design, included newborns while one study included children 0–2 years.

**Conclusions:**

Despite an extensive literature search, we did not find any studies supporting the assumption that a 20–40% bone loss is required before osteopenia becomes visible on radiographs. Reference values for cortical thickness were sparse. Further studies addressing this important topic are warranted.

## Introduction

Osteoporosis is a metabolic bone disorder characterized by low bone mass and abnormal bone architecture, resulting in bone fragility and increased susceptibility of fractures [1, 2]. In infants, exclusion of the diagnosis is crucial in cases of suspected child abuse. It is increasingly recognized that osteoporosis affects children, both as a primary problem such as osteogenesis imperfecta [3], or secondary to chronic illness, medications, diet or lifestyle issues [4, 5]. Clinical signs, if any, include a history of recurrent low impact fractures or backache, and the diagnosis is supported by the presence of risk factors and / or low bone mass on imaging. Dual energy X-ray absorptiometry (DXA) is the most widely used technique for evaluating bone mass. Bone densitometry determination is based on quantifying x-ray absorption and comparing the bone mineral density determination (BMD) with age-related reference standards. Quantification is based on a T score or the standard deviation of an individual’s BMD compared with a young, healthy reference population, matched for sex and ethnicity. In adults, a T score of less than −1 to greater than −2.5 is defined as osteopenia while a T score of −2.5 or lower is defined as osteoporosis [6].

A bone mineral density (BMD) threshold of -2.0 standard deviations (SD) or lower along with a clinically significant fracture history, is included in the International Society for Clinical Densitometry (ISCD) pediatric osteoporosis definition with one exception; the presence of a low trauma vertebral fracture, in which case the BMD threshold criteria do not apply [2, 7, 8]. Digital X-ray radiogrammetry, or the measurement of the cortical thickness of one or more metacarpal bones compared to standards, is another way to perform osteoporosis screening [9, 10].

However, whilst peak bone mass as measured by DXA [11] and digital X-ray radiogrammetry (DXR) [12] is a strong predictor of osteoporosis and fracture risk in adults, the methods have limitations in children, particularly for children under two years of age. First, the results are highly dependent on bone morphology, body size, ethnicity, pubertal staging and skeletal maturity [2]; next, normative data is sparse [13]; and third, the growing body of studies linking bone mass and childhood fracture are still not sufficiently large to establish criteria for a pediatric fracture threshold [7, 14, 15]. Although some studies have provided DXA reference data for children and adolescents of different gender, age and ethnicity [16, 17], there is a lack of data for those younger than two years of age [13, 14, 18]. These shortcomings have fueled research into alternative methods, including quantitative CT (QCT) and peripheral quantitative CT (pQCT), both of which have the advantage of measuring cortical geometry and volumetric densities of both cortical and trabecular bone, thus providing information not attainable through DXA [2]. However, neither the aforementioned CT techniques, nor new technologies such as micro-CT and high-resolution magnetic resonance imaging has been sufficiently validated in children < 2 years of age.

Recently, an automated, DXR-based method for assessing peripheral bone geometry and density has been developed, measuring a cortical index (Bone Health Index, BHI) by hand radiographs in children > 2–3 years of age [19, 20]. Although BHI assesses metacarpal cortex alone, while DXA assesses cortex and trabecular bone, several studies have demonstrated an association between the BHI and BMD as measured by DXA [21–26]. The technique builds on, and has replaced, X-ray radiogrammetry in children older than 2–3 years [10, 27], most often applied on the second metacarpal of the non-dominant hand, although other tubular bones such as the humerus, radius, clavicle, femur and tibia also have been used [9].

Given the limitations of quantitative measures, high-quality radiographs remain an option for assessment of decreased bone mass in children < 2 years of age, although this determination is subjective and based on personal experience. Moreover, both reference standards and a threshold for pathology are lacking. Irrespective of the underlying cause, the radiographic appearances of osteoporosis are those of increased radiolucency and cortical thinning [1, 2, 28]. In children the appearances of the zone of provisional calcification (ZPC) abutting the metaphysis, may provide additional information [29, 30].

According to a paper by Lachmann and Whelan in 1936 [31], later referenced by numerous authors [18, 28, 32–40], a 20–40% loss of bone mass is warranted before one can recognize osteopenia radiographically. We here report on the validity of this claim by examining the number, design and outcomes of the hitherto published studies addressing this particular issue in children 0 (term) to 2 years of age. A secondary outcome was identifying normal references for cortical thickness of the skeleton for children in the same age group.

## Materials and methods

### Search strategy

We searched the literature for publications from 1946 to January 27^th^, 2020 and amended secondly commonly referenced and highly regarded older publications. Literature searches were run, in collaboration with an experienced university librarian, in the following databases; Medline, Embase and Svemed+. Unpublished material was searched in Clinical trials and ICTRP. Both relevant subject headings and free text words were used for the following search terms: osteoporosis or osteopenia, radiography, children up to 6 years. Subject headings were adapted to the different databases (S1 Appendix). We also searched the reference lists of other relevant published reviews. We reported according to the PRISMA guidelines [41].

### Eligibility criteria

Included in the review were articles that meet accepted quality standards in relation to design and reporting, namely randomized controlled trials where available, cross-sectional, case control and cohort studies, evaluating the radiographic diagnosis of osteopenia/osteoporosis. In this setting, the term osteopenia was used as poverty of bone as assessed radiographically, while osteoporosis referred to the established ICD diagnosis. In addition, studies on reference standards should be population based and adequately sized. Studies including children aged 0–2 years in addition to other age groups were included if the actual measures could be extracted. Review articles and book chapters were identified by hand search and included to provide readers with more details.

### Study selection

All papers were exported into the Rayyan database (www.rayyan.qcri.org, free access). One examiner scored all titles/abstracts (KR). To test for agreement in the inclusion process, two additional pediatric radiologists with 14 and 10 years of experience in pediatric radiology, respectively, scored the first 1000 (JAB) and the next 500 (LSO) abstracts, as being eligible for inclusion, not eligible or uncertain. In cases of disagreement, consensus was achieved after discussion. Full agreement between two readers was reached for these first 1500 titles/abstracts, thus, the remainder titles/abstracts were scored by one author (KR) only. Papers scored as uncertain by at least one of the authors, or judged to be eligible based on the title/abstract, were read in full text and scored by two authors (KR, TAA), before a decision of inclusion was made in consensus.

### Data collection process

The following data was extracted (KR, TAA): author, journal, study year, design, study size by sex and age, number of children aged 0–2 when applicable, risk of bias, outcome measures, results. We also noted whether or not repeatability studies had been performed.

## Results

A total 5592 publications were identified, of which none met the inclusion criteria for the primary outcome, being the degree of bone loss warranted before osteopenia becomes visible radiographically.

**Fig 1:**
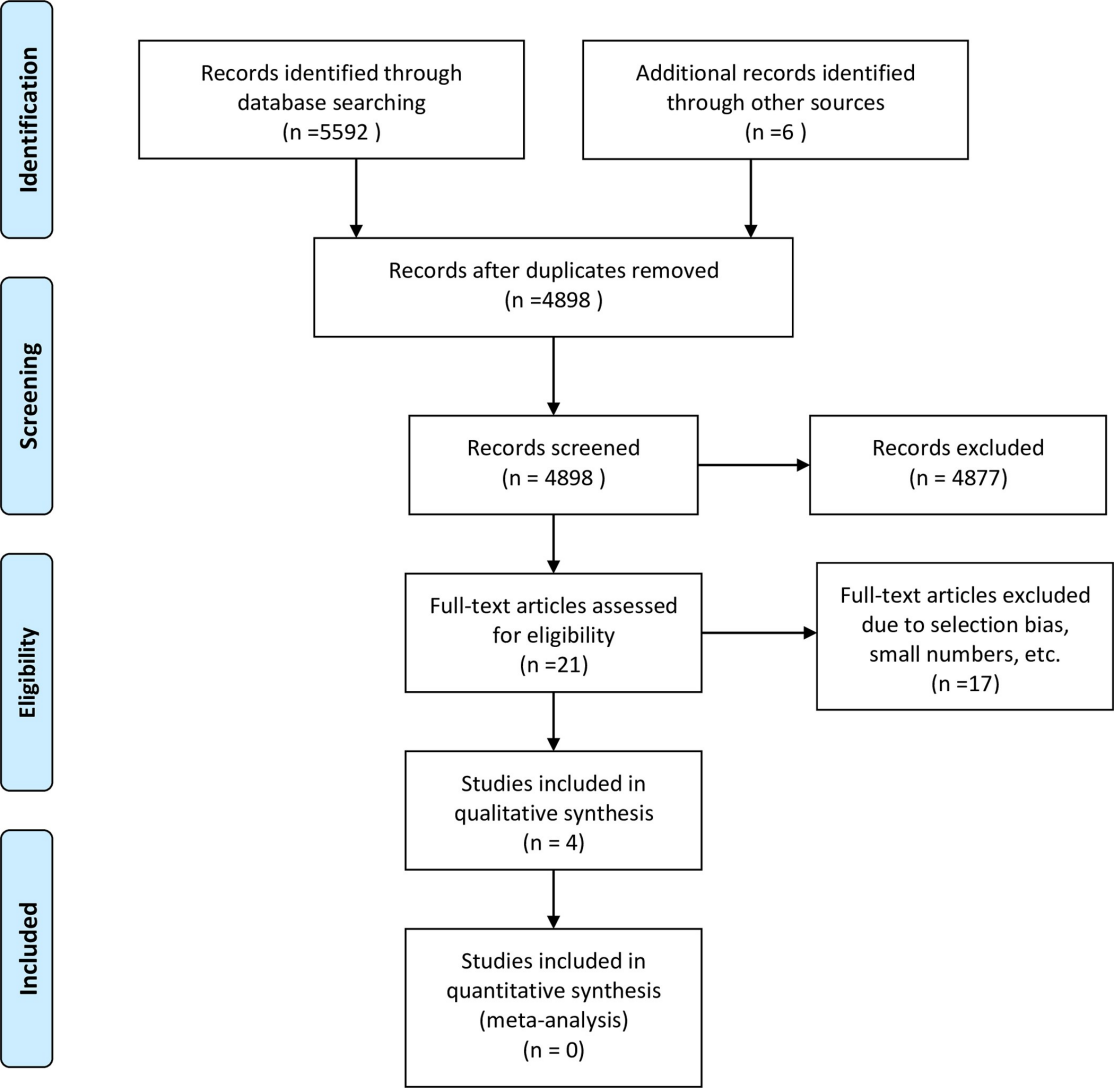
PRISMA flow Diagram of the identification process for the sample of articles addressing radiographic osteopenia or normal references for cortical thickness of the skeleton in children between 0 and 2 years of age.

As for the secondary outcome, reference standards for appearances/osteopenia in term newborns and infants < 2 years of age, 4 studies, all addressing the cortical thickness of the 2^nd^ metacarpal were included (Fig 1).

Three of the included studies were crossectional, one using a retrospective [42] and two using a prospective design [43, 44], while one was a longitudinal study [45]. None of the 4 studies covered exclusively the pre-defined age range from 0 (term) to 2 years, however, data could be extracted for the actual age group. One paper reported only the number of radiographs taken in children 0–2 years, without giving the number of children [45]. Study characteristics are given in Table 1.

**Table 1:** Reference standards for cortical thickness as measured radiographically, in healthy children under two years of age.

Title	1st author/ Year	Design (radiographs used)	Study size /age (ethnisity)	Study size, healthy, aged 0–2 yrs	Outcome measures	Potential bias	Results
Diagnosis of osteoporosis in childhood.	McCrae	Crossectional, comparison sick-healthy,	195 / 0–10 yrs	23	Metacarpal index (MI = sum of radial and cubital cortex: diameter), midpoint	Small series	Normal standards
	1967 [43]						
		(PA hand radiographs, left)				Recruited from A&E	-2^nd^ metacarpal
		Manual radiogrammetry				Precision not examined	
						No mention of sex	-0-2 yrs: mean 0.3 (SD 0.1)
Metacarpal lengths, cortical diameters and areas from the 10-state nutrition survey.	Garn	Crossectional	>10 000	40	Cortical thickness	No details on recruitment procedure	Normal standards
	1976 [44]	(hand radiographs, left)	(Caucasian)				
					(= sum of radial and cubital cortex)		-2^nd^ metacarpal
						Precision not examined	
							-1 yr: mean 1.6mm (SD 0.3) female
							-1 yr: mean 1.7mm (SD 0.5) male
Developmental changes in compact bone relationships in the second metacarpal.	Smithgall	Crossectional, retrospective	717 / 6 weeks– 26 yrs	62 (30male)	Cortical thickness	Small series	Normal standards
	1966 [42]						
		(PA hand radiographs, left)			(= sum of radial and cubital cortex), midpoint	Clinically normal, but no mention of recruitment procedur e.	-2^nd^ metacarpal
		FF = 76cm					
			(Caucasian)				
		Manual radiogrammetry					-<1 yr: mean 1.3 mm for both m/f *
						Precision inadequately examined	-1-2 yrs: mean 1.5 mm (SD 0.5)

							Minimal interobserver systematic
							error (based om 40 measurements)
Cortical thickness and diaphyseal diameter of the metacarpal bones from the age of three months to eleven years	Bonnard	Longitudinal, prospective.	175 / 0–11 yrs	538 radiographs (259 male)	Cortical thickness (= sum of radial and cubital cortex)	Number of children aged 0–2 not given	Normal standards
	1968 [45]						
			(Caucasian)				
		(hand radiographs)					-2^nd^ metacarpal
		FF = 74cm					
		No intensifying screens				Recruited from CSCG**	
							-0-2yrs: 1.1–1.3 mm (SD 0.2–0.3)
		Manual radiogrammetry					
						Precision not examined	

* = SD 0.3 for males and 0.4 for females.

** = Centre for Studies on Child Growth and Development in Zürich.

The remainder 17 articles read in full text were excluded due to small numbers [46, 47], impossible to extract results for children 0–2 years / not including this age group [27, 48–56], review [9, 14, 57], descriptive design [2, 58].

### Methods of measurements used

All 4 studies used manual radiogrammetry of the left hand, measuring the cortical thickness of the 2^nd^ metacarpal bone (sum of radial and cubital cortex), except for one using a metacarpal index (MI = cortical width x 2, divided by the diameter of the diaphysis, 2^nd^ metacarpal) [43].

### Potential bias

Although the total study sizes were high for all papers, the number of children under the age of two was small, ranging from 23–62 children, a total of 94 children. Two studies reported on technical details such as film-focus distance, ranging from 74-76cm [42, 45]. All studies included Caucasians. Two of the four studies reported on how the children had been recruited, namely from the A&E-department, attending for trivial complaints [43] or from the Centre for Studies on Child Growth and Development in Zürich [45].

### Precision

Only one of the studies had examined repeatability (precision) of the measurements [42], by performing a check on 40 randomly selected x-rays showing no systematic inter-observer error, with no mention of statistical analysis used.

### Summarized results

Based on 3 studies [42, 44, 45], the mean cortical thickness of the 2^nd^ metacarpal ranged from 1.1 to 1,7mm, with a standard deviation from 0,2 to 0.5mm. None reported on significant differences according to sex.

## Discussion

Despite an extensive, systematic literature search, we found no evidence for the assumption that there has to be a 20–40% loss of bone mass before osteopenia is evident radiographically. References for cortical thickness included 4 studies of the 2^nd^ metacarpal, and were based on small datasets with no robust analysis of measurement precision.

**Fig 2:**
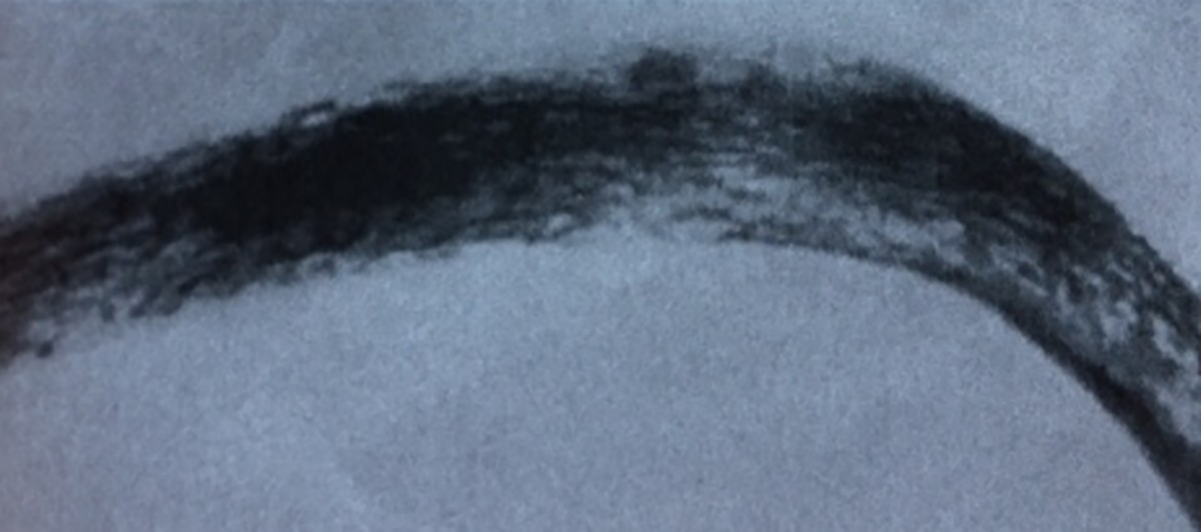
Radiograph of an artificially decalcified rib, with 54.7% of the calcium removed. From: Lachman E and Whelan M.A: The roentgen diagnosis of osteoporosis and its limitations. Radiology 26, 165–177 (1936) (with permission).

In their original work from 1936, Lachmann and Whelan [31] included one adult bone (specimens) from 10 different locations, including rib, skull, scapula, head- diaphysis and distal end of humerus, calcaneus, metacarpal, navicular and vertebra. Where possible, they used a pair of bones; one for their experiment and the other serving as a control. The bones were treated in 10% nitric acid for decalcification, before radiographs were taken. Finally, the bones were ashed, and the loss of minerals weighed. However, the quality of the radiographs from 1936 were indeed poor as compared to current standards (Fig 2). The authors reported a first faint, and a second distinct appearance of osteoporosis after varying degrees of de-calcification for each of the bones. Faint osteoporosis was, for instance, detected after around 7% calcium loss of the head of the humerus and the calcaneus, while distinct osteoporosis was seen after 16% and 14% calcium loss, respectively. For the vertebra, corresponding figures were around 8% and 14%. In contrast, calcium loss of up to 40% was judged necessary for visualization of distinct osteoporosis in the distal humerus. The results of these experiments have repeatedly been referred to for the past 80 years [1, 8, 13, 18, 28, 32, 33], although the study design did not allow any conclusions to be drawn. In sum, there is *no* literature to support this assumption.

The death of the abovementioned myth, and the lack of robust reference standards for cortical thickness leaves us with a subjective, experience-based approach for identification of decreased bone mineral density in children born term, under the age of two. Increased radiolucency is the result of resorption and thinning of the trabeculae, and the term *osteopenia* (“poverty of bone”) has been used as a generic designation for radiographic signs of decreased bone density in adults [1]. Irrespective of the underlying cause, the radiographic appearances of osteopenia are those of increased radiolucency and cortical thinning [1, 2, 28]. In young children, analysis of the radiographs and specifically the metaphyses of long bones, suggests the origin of decreased bone mineral density, and hence we can replace the term osteopenia for more specific terms such as osteoporosis or rickets [59].

In the immature skeleton of the fetus, infant and child, bone mineralization occurs through two processes, endochondral ossification where the cartilaginous matrix of the lower physis becomes increasingly calcified, and intramembranous ossification, where there is formation of osteoblasts which secrete osteoid, an unmineralized matrix that subsequently calcifies [60]. Abnormalities of osteoid synthesis or mineralization result in osteoporosis. A typical example is osteogenesis imperfecta, where abnormal type 1 collagen creates an abnormal osteoid.

**Fig 3:**
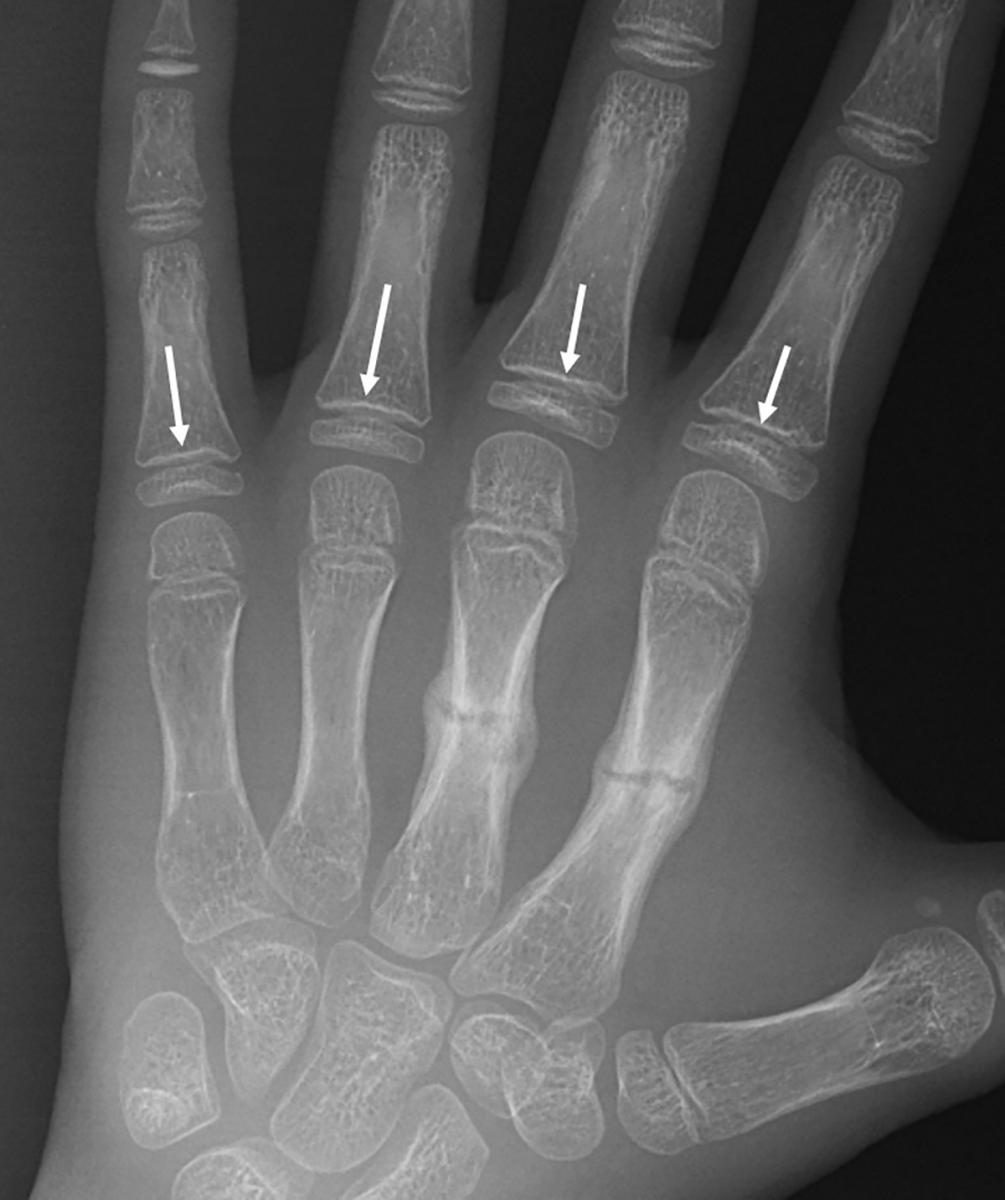
Radiograph of the hand in a 12-year-old boy with healing fractures to the 2^nd^ and 3^rd^ metacarpals, showing osteopenia due to inactivity. The zone of provisional calcification becomes more conspicuous, as its visualization is enhanced by the radiolucency in the metaphyseal side (arrows).

Abnormalities of endochondral ossification, exemplified by rickets, result in a poorly differentiated or absent zone of provisional calcification. The zone of provisional calcification (ZPC) is considered a marker for bone health in children until closure of the physis [29, 30]. Specifically, an intact zone of provisional calcification indicates that there is a healthy process of endochondral ossification. Radiographically, the ZPC is seen as a sharply defined, transverse radiodense band at the chondro-osseous junction [61]. Lack of calcium, vitamin D or phosphate all interfere with the endochondral ossification process, and result in loss/indistinctness of the radiologic ZPC [29, 62]. In contrast, when there is rapid loss of bone mass, as in disuse osteoporosis or in neonates in the intensive care unit, bone loss begins in the adjacent highly vascularized metaphyseal spongiosa. The zone of provisional calcification becomes more conspicuous, as its visualization is enhanced by the radiolucency in the metaphyseal side (Fig 3). The role of the zone of provisional calcification and metaphyseal spongiosa in assessing decreased bone mineral density needs, however, further studies.

Trabecular bone responds to metabolic changes faster than does cortical bone [63], and changes are most prominent in the axial skeleton and in the ends of the long tubular bones, particularly in the proximal femur and distal radius as these have a relatively large proportion of trabecular bone.

In contrast to extremity bones, vertebrae contain a higher proportion of trabecular bone, which is more metabolically active than cortical bone and thus more exposed to the osteotoxic effect of drugs such as glucocorticoids. Not all vertebrae are equally vulnerable, with most fractures in children located in the upper thoracic (T6/7) and lumbosacral (L1/2) spine [64].

A limitation to our study relates to the lack of available studies, hindering a structured report according to the PRISMA guidelines. The strengths include the wide, extensive literature search performed together with an experienced university librarian and the meticulous process for selecting studies. To minimize bias during identification of eligible papers, the title/abstract of the first 1500 articles were screened independently by two authors. Full agreement was reached in all 1500, thus, we felt that the remainder of the abstracts were safely scored by one of the authors only, without introducing major bias.

## Conclusion

An extensive systematic review of the literature did not support the assumption that there has to be a 20–40% loss of bone mass before osteopenia is evident radiographically. Moreover, the references for cortical thickness were based on small datasets with no valid analysis of observer agreement. Further studies addressing this important topic are warranted, as the diagnosis of osteopenia is of utmost importance in this particular age group.

## Supporting information

S1 Checklist PRISMA 2009 checklist. (DOC)

S1 Appendix Search strategies. (DOC)
