# Supplementary material for: Revisiting the radiographic assessment of osteoporosis—Osteopenia in children 0–2 years of age. A systematic review
Source: PLoS One. 2020 Nov 2;15(11):e0241635. doi: 10.1371/journal.pone.0241635 (PMC7605664; doi:10.1371/journal.pone.0241635)
Supplement: S1 Appendix — (DOC) [file pone.0241635.s002.doc]

# S1 Appendix.

In September 2018 (with follow-up searches in November 2019 and in January 2020) we searched Medline (Ovid), Embase (Ovid), and the Scandinavian database Svemed+ for published studies, and Clinical trials.gov and **International Clinical Trials Registry Platform Search Portal** for unpublished studies. The search terms covered the concepts of osteopenia or osteporosis, radiography, and children. The searches were run with a combination of text words and subject headings, adapted to each database, without any date or language limits. Detailed search strategies for the latest search dates are shown.

The results of the searches were pooled. We also searched the reference lists of articles identified by this search strategy and selected those we judged relevant.

**Database: Ovid MEDLINE(R) and Epub Ahead of Print, In-Process & Other Non-Indexed Citations and Daily <1946 to January 27, 2020> Searched at 2020-jan-28**

1 exp Bone Diseases, Metabolic/ (75401)

2 Bone Density/ (52246)

3 exp Cortical Bone/ (1600)

4 (osteoporosis or osteopenia or bone density or Bone Deminerali?ation or Decalcification or Pseudohypoparathyroidism or Pseudopseudohypoparathyroidism or Rickets or ("Chronic Kidney Disease" adj3 "Mineral and Bone Disorder") or Osteomalacia).ti,ab,kw. (95458)

5 ((cortical or complex or Cancellous or trabecular or spongy) adj1 (bone or bones)).ti,ab,kw. (30136)

6 1 or 2 or 3 or 4 or 5 (160926)

7 exp RADIOGRAPHY/ (1091852)

8 diagnostic imaging.fs. (1154347)

9 (radiograph* or radiogram* or radiology or roentgen* or x ray* or xray*).ti,ab,kw. (627049)

10 7 or 8 or 9 (1911184)

11 6 and 10 (49446)

12 child, preschool/ or exp infant/ (1543742)

13 (newborn or baby or infant* or preschool* child).ti,ab,kw. (522942)

14 12 or 13 (1709922)

15 11 and 14 (3296)

**Database: Embase (OVID) <1974 to 2019 November 01> Searched at 2019-nov-04**

1 exp metabolic bone disease/ (154233)

2 bone density/ (90203)

3 cortical bone/ (17591)

4 (osteoporosis or osteopenia or bone density or Bone Deminerali?ation or Decalcification or Pseudohypoparathyroidism or Pseudopseudohypoparathyroidism or Rickets or ("Chronic Kidney Disease" adj3 "Mineral and Bone Disorder") or Osteomalacia).ti,ab,kw. (141398)

5 ((cortical or complex or Cancellous or trabecular or spongy) adj1 (bone or bones)).ti,ab,kw. (39862)

6 trabecular bone/ (20616)

7 1 or 2 or 3 or 4 or 5 or 6 (256388)

8 exp bone radiography/ (69661)

9 (radiograph* or radiogram* or radiology or roentgen* or x ray* or xray*).ti,ab,kw. (721021)

10 8 or 9 (757309)

11 7 and 10 (46425)

12 exp infant/ (954378)

13 preschool child/ (527998)

14 (newborn or baby or infant* or preschool* child).ti,ab,kw. (582111)

15 12 or 13 or 14 (1480225)

16 11 and 15 (2290)

**Svemed+ (Karolinska institutet), (from 1977- ) Searched at 4 Nov 2019**

1 exp:"Bone Diseases, Metabolic" 762

2 exp:"Bone Density" 489

3 exp:"Cortical bone" 0

4 osteoporosis OR osteopenia 772

5 #1 OR #2 OR #3 OR #4 950

6 exp:"RADIOGRAPHY" 4939

7 exp:"Child, Preschool" 3907

8 exp:"Infant" 5630

9 #7 OR #8 7508

10 #5 AND #6 AND #9 6

**Clinical Trials:** [**https://clinicaltrials.gov/**](https://clinicaltrials.gov/) **Searched at 4 Nov 2019**

**Condition or disease: Osteoporosis OR osteopenia**

**Other terms: radiography AND (preschool child OR infant)**

**6 studies**

**International Clinical Trials Registry Platform Search Portal - WHO-ICTRP:** [**http://apps.who.int/trialsearch/**](http://apps.who.int/trialsearch/) **Searched at 4 Nov 2019**

**Search in title: (Osteoporosis OR osteopenia) AND radiograph***

**Limit to “Clinical trials in children”**

0 records for 0 trials found
